# The Role of MRI Radiomics Using T2-Weighted Images and the Apparent Diffusion Coefficient Map for Discriminating Between Warthin’s Tumors and Malignant Parotid Gland Tumors

**DOI:** 10.3390/cancers17040620

**Published:** 2025-02-12

**Authors:** Delia Doris Donci, Carolina Solomon, Mihaela Băciuț, Cristian Dinu, Sebastian Stoia, Georgeta Mihaela Rusu, Csaba Csutak, Lavinia Manuela Lenghel, Anca Ciurea

**Affiliations:** 1 Radiology and Imaging Department, Iuliu Hațieganu University of Medicine and Pharmacy, 400012 Cluj-Napoca, Romania; 2 Oro-Maxillo-Facial Surgery Department, Iuliu Hațieganu University of Medicine and Pharmacy, 400012 Cluj-Napoca, Romania

**Keywords:** MRI, radiomics, textural analysis, parotid gland tumors, Warthin’s tumors, malignant tumors

## Abstract

Background/Objectives: Differentiating between benign and malignant parotid gland tumors (PGT) is essential for establishing the treatment strategy, which is greatly influenced by the tumor’s histology. The objective of this study was to evaluate the role of MRI-based radiomics in the differentiation between Warthin’s tumors (WT) and malignant tumors (MT), two entities that proved to present overlapping imaging features on conventional and functional MRI sequences. Methods: In this retrospective study, a total of 106 PGT (66 WT, 40 MT) with confirmed histology were eligible for radiomic analysis, which were randomly split into a training group (79 PGT; 49 WT; 30 MT) and a testing group (27 PGT; 17 WT, 10 MT). The radiomic features were extracted from 3D segmentations of PGT performed on the following sequences: PROPELLER T2-weighted images and the ADC map, using a dedicated software. First- and second-order features were derived for each lesion, using original and filtered images. Results: After employing several feature reduction techniques, including LASSO regression, three final radiomic parameters were identified to be the most significant in distinguishing between the two studied groups, with fair AUC values that ranged between 0.703 and 0.767. All three radiomic features were used to construct a Radiomic Score that presented the highest diagnostic performance in distinguishing between WT and MT, achieving an AUC of 0.785 in the training set, and 0.741 in the testing set. Conclusions: MRI-based radiomic features have the potential to serve as promising novel imaging biomarkers for discriminating between Warthin’s tumors and malignant tumors in the parotid gland. Nevertheless, it is still to prove how radiomic features can consistently achieve higher diagnostic performance, and if they can outperform alternative imaging methods, ideally in larger, multicentric studies.

## Simple Summary

Radiomics is an innovative quantitative post-processing imaging technique that enables the extraction and analysis of textural information from medical images that are not discernible through simple visual assessment. The current gold standard imaging method to diagnose parotid gland tumors is the multiparametric MRI. However, there are still significant overlapping MRI features between various tumor types, especially between Warthin’s tumor and malignant tumors. Therefore, this study aims to assess the potential role of MRI radiomics in this current issue.

## 1. Introduction

Salivary gland tumors are infrequent, accounting for approximately 2–6.5% of all head and neck lesions [1,2]. The World Health Organization (WHO) identified a total of 38 salivary gland tumor types, of which 23 are malignant [3]. Most of the tumors occur in the parotid gland, in about 80% of cases, of which 80% are benign and 20% malignant [4]. As current imaging techniques cannot correctly diagnose all of the salivary gland tumor types, the essential is to determine at least whether a salivary gland lesion is benign or malignant, as it influences the therapeutic approach and dictates the type of surgical resection required.

For parotid gland tumors (PGTs), the gold standard of treatment is surgery. Local resection or partial parotidectomy is, in most cases, sufficient for benign lesions, whereas malignant lesions often require total parotidectomy at times also followed by chemoradiotherapy [5,6]. Therefore, accurate preoperative diagnosis is critical for selecting the appropriate treatment and ensuring optimal patient outcomes.

Core needle biopsy and fine needle aspiration cytology are regarded as reliable methods for diagnosing salivary gland neoplasms. Nevertheless, their diagnostic accuracy may be compromised at times due to overlapping histological or cytological patterns between benign and malignant PGT, and also due to sampling errors. Additionally, PGTs localized in the deep lobe are not always feasible for tissue sampling [7]. Parotid gland biopsy is generally considered a safe procedure; however, rare complications have been reported such as hematoma, seroma, infections, and facial nerve paralysis [8], which could be avoided with the use of reliable non-invasive diagnostic imaging techniques.

The gold standard imaging method for assessing PGTs is multiparametric MRI, which provides both morphological and functional information [9,10,11]. Several multiparametric flowcharts have been designed to aid in diagnosing the most common types of PGTs. The most common included MRI features were the tumor’s margins, the T1 and T2-weighted images signal intensity, the apparent diffusion coefficient (ADC) value, and the type of time-intensity curve obtained using dynamic contrast-enhanced perfusion-weighted imaging [10,12]. However, there are still significant overlapping MRI patterns between different PGTs.

Warthin’s tumors (WTs), although benign, share certain features with malignant tumors (MTs), which can sometimes pose diagnostic difficulties. Both WTs and MTs can show heterogeneity due to their cystic and solid components, low ADC values, and overlapping enhancement behavior [11,13]. Even in PET scans, WTs can demonstrate high FDG uptake, similar to malignant tumors, potentially leading to false-positive results. Furthermore, WT can present bilaterally or multifocally, features also seen in some MTs, particularly metastases [14]. Therefore, identifying the alternative noninvasive imaging techniques that could improve the accuracy of preoperative diagnosis of WTs and MTs remains a significant challenge.

Radiomics represents a new quantitative postprocessing technique of medical images that allows the quantification of textural information that cannot be assimilated by simple visual inspection of the images. The fundamental hypothesis of radiomics is that images reflect the phenotypic expression of underlying biological processes. Therefore, by extracting textural quantitative parameters, the “composition” of the pixels in one image can be assessed [15].

In the era of personalized medicine, radiomics has garnered significant interest, and has emerged as a promising complementary diagnostic tool, offering potential support in clinical decision-making across various pathologies, including those involving the salivary glands [16]. Most studies published so far mitigated the textural analysis input in differentiating between benign and malignant PGTs, or between pleomorphic adenoma and WTs, as these are the most commonly encountered PGTs [17,18], whereas differentiating between WTs and MTs has been addressed in only a few studies, and needs further validation [19,20]. Therefore, this study sought to evaluate the role of MRI radiomics in differentiating between WTs and MTs in the parotid gland, using 3D tumor segmentations performed on T2-weighted images (T2-WI) and the ADC map.

## 2. Materials and Methods

This retrospective study was granted approval by the local ethical committee (date: 11th of February 2022; registration number: 43) and the requirement for obtaining informed consent from patients was waived.

An electronic scrutiny of the Picture Archiving and Communication System (PACS) and Hospital Information System (HIS) was conducted in order to identify patients with parotid gland lesions who underwent head–neck MRI examinations at our referral imaging center between January 2018 and October 2024. This study adhered to the essential principles outlined in the Checklist for Evaluation of radiomics research (CLEAR) guidelines to ensure methodological rigor and transparency in the research process [21].

### 2.1. Inclusion and Exclusion Criteria

The inclusion criteria were defined as follows: (a) patients with a histological confirmation (by biopsy or surgical material) of WT and MT (in the latter category, only primary epithelial malignant tumors and secondary tumors were included); (b) patients who had undergone MRI examinations that included T2-WI PROPELLER and Diffusion-Weighted Imaging (DWI) with ADC maps; and (c) in patients with multiple lesions, only lesions with a diameter greater than 1 cm were included. The exclusion criteria were: (a) patients younger than 18 years old; (b) MRI examinations performed after biopsy or any surgical procedure; (c) MRI sequences with impaired image quality due to motion or metallic artifacts; (d) parotid lesions < 1 cm; and (e) lymphomatous parotid lesions. Studies consistently show that lymphoma is characterized by high cellular density, which leads to restricted diffusion and significantly low and pathognomonic ADC values (ranging from 0.4 to 0.7 × 10^−3^ mm^2^/s) [11,12]. Consequently, we excluded this tumor type to avoid any potential bias in differentiating MTs from WTs.

There were 79 consecutive patients that matched the inclusion and exclusion criteria (28 female, 51 male, median age 61, range 35–80). Since 20 patients presented multiple parotid gland lesions of the same histopathological type, for this study, a final number of 106 PGTs (66 WTs, 40 MTs) were eligible for the radiomic analysis. Details regarding the tumor distributions are shown in Table 1.

The samples were randomly allocated to a training group (79 PGTs; 49 WTs; 30 MTs) and a testing group (27 PGTs; 17 WTs, 10 MTs), according to the “one-third” criteria recommended in radiomic studies [22].

### 2.2. Image Acquisition

All MRI examinations were carried out in our imaging center on a commercial 1.5-Tesla MRI scanner (SIGNA™ Explorer, General Electric, Milwaukee, WI, USA) with a dedicated, 16-channel, head and neck coil. A standardized MRI protocol for assessing PGTs was used, which included: axial fast spin echo T2-WI Periodically Rotated Overlapping Parallel Lines with Enhanced Reconstruction (PROPELLER); axial fast spin echo T1-WI with and without fat saturation; coronal short tau inversion recovery (STIR), axial echo-planar DWI at multiple b-values with the resulting apparent diffusion coefficient (ADC) map; axial perfusion-weighted imaging; and an axial fast spin echo fat-saturated contrast-enhanced T1-WI using intravenous contrast medium 0.1 mL/kg Gadobudrol, (Gadovist; Bayer HealthCare, Berlin, Germany).

For the texture analysis, the following sequences were used:

- T2-WI PROPELLER: echo time = 80 ms; repetition time = 5500 ms; slice thickness = 3 mm; slice gap = 3.6 mm; flip angle = 160°, matrix (mm) 384 × 384.
- ADC map automatically generated by the MRI vendor from the DWI: b-values = 0 and 1000 s/mm^2^; echo time = 100 ms; repetition time = 6800 ms; slice thickness = 3 mm; slice gap = 3.6 mm; flip angle = 160°, matrix (mm) 384 × 384.

The MRI sequences were retrieved in DICOM format following data anonymization.

The T2-WI sequence was chosen, as it is particularly sensitive to water content and tissue structural details, often providing clear signal intensity contrast between tumors and the neighboring normal tissue [18,19]. The ADC map offers an insight into tumoral cellular density as a cause of heterogeneity [Fruemar], and it was chosen as several studies reported that the overlapping ADC values between PGT types could be mitigated through whole-tumor analysis on the ADC map [23,24].

### 2.3. Tumor Segmentation

**Figure 1:**
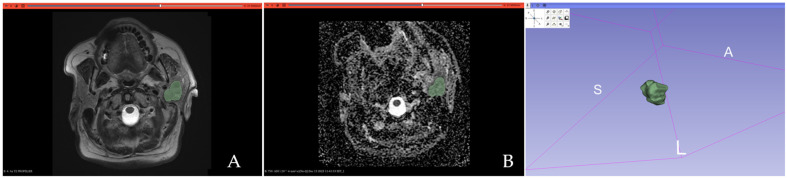
Three-dimensional segmentation of a malignant parotid gland tumor (histopathologically confirmed acinic cell carcinoma) performed on the T2-weighted image (**A**) and the ADC map (**B**).

**Figure 2:**
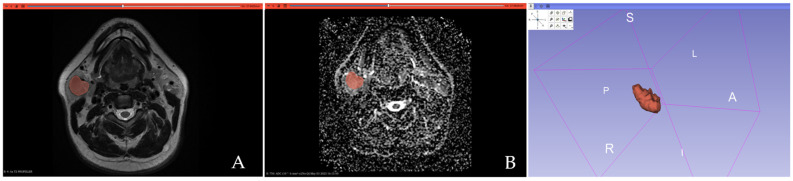
Three-dimensional segmentation exemplification of a histopathologically confirmed Warthin’s tumor performed on the T2-weighted image (**A**) and the ADC map (**B**).

All PGTs were manually segmented by one radiologist with 5 years of experience in head and neck imaging, blinded to the histopathological results, using open-source textural analysis software 3D-Slicer (version 4.14, available online at: http://www.slicer.org/ accessed on 1 August 2024). A 3D segmentation was first performed on T2-WI by manually outlining each lesion slice by slice, aiming to encompass the tumor’s maximum area without exceeding its boundaries. Necrotic areas and vessels were excluded. The resulting 3D segmentations were then automatically applied to the ADC maps. Figure 1 and Figure 2 show examples of MTs and WT 3D segmentations on the T2-WI and the ADC map.

### 2.4. Image Preprocessing

All images underwent Z-score normalization and discretization using a fixed-bin width of 25. To correct the spatial signal variability due to inherent MRI field inhomogeneities, the N4ITK bias field correction was applied [25]. The well-established “µ ± 3σ” method was utilized to mitigate the effects of variable protocol parameters from different MRI scanners. This allowed for the identification and removal of image intensity outliers that deviated >3 sigma from the mean [26]. As some radiomic feature values proved to be dependent on voxel size, a linear interpolator (B-Spline) was used to resample all images to a voxel size of 1 × 1 × 1 mm^3^ [27]. For extracting the 3D textural features, isotropic resampling is preferred over anisotropic resampling, as it ensures uniformity in orientation and scaling [28]. Preprocessing filters were also used, such as wavelet and Laplacian of Gaussian (LoG) with coarse patterns (sigm 5.0 mm) and fine patterns (sigma 3.0 mm). Wavelet filters discriminate between low and high spatial frequency information, while LoG filters can highlight regions undergoing fast changes (such as edge detection) [29].

### 2.5. Feature Extraction

From the PGT 3D segmentation on each MRI sequence, a total of 1037 quantitative radiomic parameters were retrieved from both original and filtered images, using the open-source PyRadiomics package (version 3.0.1, available at https://pyradiomics.readthedocs.io/en/latest/, accessed on 1 September 2024). The extracted radiomic features categories are presented in Table 2.

### 2.6. Feature Selection and Statistical Analysis

To assess feature stability, 30 lesions (15 WTs, 15 MTs) were randomly selected and manually resegmented by the same researcher two weeks after the first segmentation. The intraclass correlation coefficient (ICC) was used to assess the intraobserver agreement, and only features with an ICC > 0.85 were further included in the analysis.

Before feature selection, all radiomic quantitative features were standardized using z-score normalization. To minimize overfitting and reduce bias from radiomic features in the modeling process, several feature reduction steps were conducted in the training dataset.

The Mann–Whitney U univariate test with the Benjamini–Hochberg correction for multiple testing was performed to determine statistically significant radiomic features able to distinguish between WT and MT. Features with an adjusted *p*-value < 0.05 were enrolled for the next step, and the Spearman correlation was assessed between any two features. To reduce redundancy, when a pair of highly correlating features was identified (Spearman’s coefficients <−0.9 or >0.9), the feature with the highest *p*-value in the univariate analysis was eliminated.

The least absolute shrinkage and selection operator (LASSO) regression algorithm was used as a last approach for dimensionality shrinkage. The minimum criteria were applied to optimize the regularization parameter (λ) for feature selection through 10-fold cross-validation. The most predictive radiomic parameters with non-zero coefficients were combined into a Radiomic Score calculated by weighting the coefficients based on the LASSO logistic regression model.

The receiver operating characteristic (ROC) curve analysis was performed to assess the diagnostic performance of the final selected radiomic features and the developed Radiomic Score.

The statistical analysis was conducted using MedCalc (version 23.0.9), SPSS Statistics for Windows (version 18.0), and R software (version 3.6.3 with the “glmnet” package).

**Figure 3:**
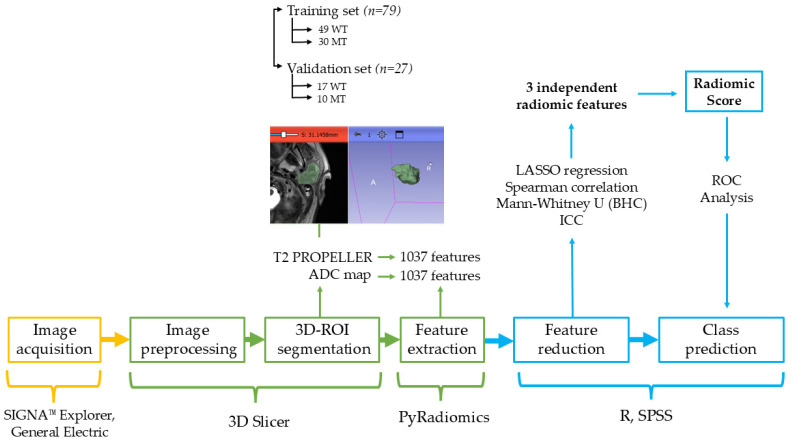
The radiomic pipeline. WT = Warthin’s tumors; MT = malignant tumors; ICC = intraclass correlation coefficient; BHC = Benjamani–Hochberg Correction; LASSO = least absolute shrinkage and selection operator; ROC = receiver-operating characteristic.

The radiomic pipeline performed in this study is presented in Figure 3.

## 3. Results

A total of 79 patients (28 females, 51 males; median age 61, range 35–80) were included in the study. As 20 patients had multiple parotid gland lesions of the same histopathological type, a total of 106 PGT (66 WT, 40 MT) were eligible for the radiomic analysis in this study.

### 3.1. Feature Selection Results

**Figure 4:**
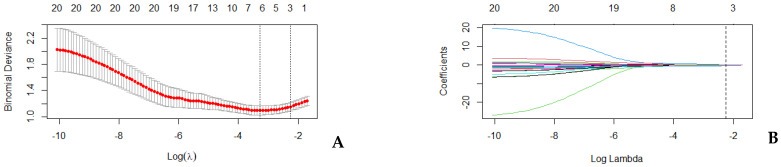
LASSO regression. (**A**) Cross-validation curve: *X*-axis represents the logarithm of the regularization parameter lambda (*λ*); *Y*-axis represents the binomial deviance as a measure of model fit; the red dots represent the mean binomial deviance calculated for each value of *λ* during cross-validation; the error bars show the standard error of the binomial deviance at each *λ*; the first vertical line corresponds to the *λ* value that minimizes the deviance (the optimal *λ*; the second vertical line represents the largest *λ* within one standard error of the minimum deviance. (**B**) Coefficient path: The *X*-axis shows the logarithm of the regularization parameter *λ*; the *Y*-axis represents the magnitude of the regression coefficients; the colored lines represent the paths of individual coefficients as *λ* changes; the vertical dotted line corresponds to the optimal *λ* selected by cross-validation.

From the 3D PGT segmentation in the training set, 1037 features were extracted from each MRI sequence. After assessing the intrareader variability, 840 T2-WI features and 700 ADC features, with ICC > 0.85, were retained. The univariate analysis found 90 T2-WI features and 76 ADC features that could differentiate between the two studied groups with statistical significance. Spearman’s correlation revealed 20 non-redundant features (Table 3) that were further included in the LASSO regression model with 10-fold crossed validation (Figure 4).

### 3.2. Diagnostic Accuracy of the Final Selected Radiomic Parameters

Following LASSO regression, three final radiomic parameters were identified to be the most significant in distinguishing between WT and MT (Table 4), which presented fair AUC values ranging between 0.703 and 0.767 (Table 5).

### 3.3. The Diagnostic Accuracy of the Radiomic Score in the Training and Validation Datasets

The three most predictive radiomic features were linearly combined using their corresponding LASSO coefficients to generate a Radiomic Score, using the following formula:

(1) $$Radiomic\;Score=I+\sum_{y=0}^{3}C_{y}*\;V_{y}$$

where ***I*** represents the intercept, ***Cy*** the coefficient of the ***y***th radiomic parameter, and ***Vy*** the numerical value of the ***y***th radiomic parameter.

The Radiomic Score showed statistically significant higher values for WTs in comparison to MTs, in both the training set and testing set. The median Radiomic Score value for WTs and WTs in the training set was 0.33 [−0.26 to 0.83] vs. −0.75 [−1.07 to −0.30], *p* < 0.001, while in the testing set was 0.12 [−0.56 to 0.78] vs. −0.88 [−1.41 to −0.23], *p* = 0.032, respectively.

**Figure 5:**
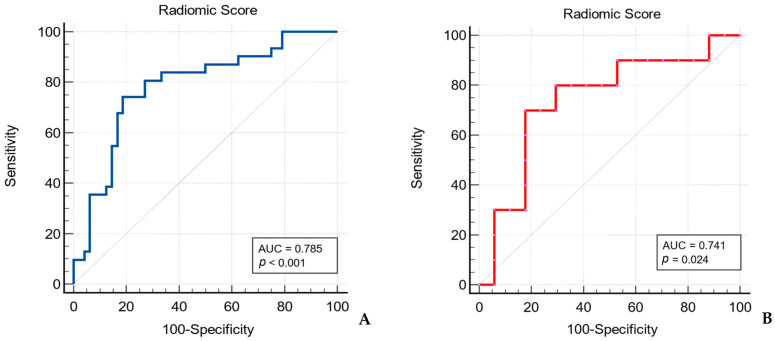
Receiver operating characteristic (ROC) curve of the Radiomic Score for differentiating between Warthin’s tumors and malignant tumors of the parotid gland in the training set (**A**) and testing set (**B**).

The Radiomic Score presented higher diagnostic performance in distinguishing between WTs and MTs in comparison to each radiomic feature alone, achieving an AUC value of 0.785, with 74.19% sensitivity and 81.25% specificity (*p* < 0.001). When applied to the testing set, the Radiomic Score reached an AUC value of 0.741 (Table 6, Figure 5).

## 4. Discussion

The clinical need for better diagnosis and management has led to an expanding interest in applying radiomics in many medical fields. As there are still overlapping imaging patterns between different PGT on both conventional and functional MRI sequences, radiomics, and textural analysis might represent a new tool in discovering additional biomarkers that could increase the diagnostic accuracy in this pathology [16].

Several radiomic studies focused on differentiating between PGT, by extracting textural features from either single or multiple MRI sequences and proposed radiomic models with variable diagnostic performance [30,31]. However, a widely accepted and consistently validated radiomic signature has yet to be established in the literature.

In this study, we investigated the radiomics performance based on T2-WI PROPELLER and the ADC maps in differentiating between WT and MT in the parotid gland. We selected T2-WI PROPELLER, as it reflects heterogeneity due to different tissue water content and can reveal the inner structure of tumors in greater detail than T1-WI [32]. Furthermore, the PROPELLER technique enhances image quality and reduces the likelihood of motion artifacts [33]. DWI assesses the Brownian motion of water molecules between intracellular and extracellular compartments of biological tissues, reflecting their microanatomy. The corresponding apparent diffusion coefficient (ADC) reflects the hydrogen proton movement within tissues, which proved to be restricted in tissues with high cellularity such as tumors, reflecting therefore tissue heterogeneity in the degree of cellular packing [34]. Also, both sequences are part of the standard head–neck MRI exam, and can be obtained without the routine use of contrast agents.

We performed 3D segmentations of all PGTs, as we considered it would offer a more comprehensive heterogeneity assessment in comparison to a 2D approach.

The constructed Radiomic Score in this study included radiomic features extracted from filtered images: two second-order radiomic features (derived from gray-level size zone matrix and gray-level run length matrix) and one first-order radiomic feature. The Radiomic Score achieved an AUC value of 0.785 in the training set, greater than the AUC reached by each radiomic parameter separately, which varied between 0.702 and 0.767. The Radiomic Score presented an AUC value of 0.741 in the testing dataset.

So far, there are few studies published in the literature that specifically addressed the radiomic contribution in differentiating between WTs and MTs of the parotid gland. The proposed radiomic models presented variable diagnostic performances.

Gabelloni et al. [19] identified two radiomic parameters derived from T2-WI, cluster prominence value and the sum of square variance value, that proved to be the most discriminative between WTs and MTs, with an AUROC of 0.714 and 0.711, respectively. A support vector machine classifier was trained using individual features as well as their combination. The highest classification performance for differentiating between the two groups was achieved with the cluster prominence value. However, the results were suboptimal, with low sensitivity and specificity values of 0.621.

The linear discriminant analysis model based on ADC radiomic features proposed by Wen et al. presented even lower diagnostic performance, reaching an AUC value of 0.592 [20].

Conversely, more promising results were obtained in one study that assessed the diagnostic performance of first-order radiomic features extracted from fat-saturated contrast-enhanced T1-WI and T2-WI in differentiating between WTs and a specific MT type: mucoepidermoid carcinoma. The results showed that kurtosis on T2-WI reached the largest AUROC (0.788), with a sensitivity of 80% and specificity of 77.8% [35].

Another study identified three ADC histogram parameters significantly different between WTs and MTs. The best diagnostic performance was achieved by skewness, followed by ADC_50th and ADC_mean with AUC of 0.782, 0.737, and 0.724, respectively [23].

The Radiomic Score in this current study presented a fair diagnostic performance (AUC of 0.785) in differentiating between WTs and MTs of the parotid gland.

We consider that further research is necessary to develop even better classification algorithms and radiomic signatures, as there is yet only fair diagnostic accuracy generally reported in MRI textural analysis studies particularly focused on discriminating WT from MT [19,20,23,35].

This study presents several limitations that need to be discussed. Firstly, there was a small sample size included in the MT group. However, this is in accordance with the reported prevalence of malignancies affecting the parotid gland [36]. Lymphomatous parotid lesions were excluded to avoid any bias, given the consistent evidence in studies that lymphoma presents with high cellular packing that generates restricted diffusion with extremely low and pathognomonic ADC values between 0.4−0.7 × 10^−3^ mm^2^/s [11,12]. The fact that we considered all of the included malignant tumors as a single entity is also a limitation, given the fact that they might show different imaging features related to specific histological types, and therefore represent a source of heterogeneity.

As suggested in radiomic studies, we divided the available data into training and testing datasets. However, we could not validate the Radiomic Score in an independent external set.

Another important limitation is that the Radiomic Score was not compared to clinical–radiological models or complex radiomic–clinical–radiological models. The low number of malignant cases in the study limited the ability to perform a fair multivariate logistic regression analysis and reach statistical significance for both training and testing datasets.

MR images exhibit significant signal intensity variations between examinations, which represent a major challenge in radiomic studies that require particular focus [37]. Even though all patients were scanned using a standardized head–neck MRI protocol, variations in acquisition parameters might still occur. To address this, several pre-processing techniques were applied to the MR images before feature extraction to ensure a homogeneous dataset.

Another critical consideration is that many textural analysis software extract a vast number of radiomic features, which often exceed the sample size and increase the risk of overfitting [38]. To mitigate this, we employed multiple-step feature reduction techniques to eliminate redundant radiomic parameters. Similar approaches were also conducted in earlier radiomic studies with promising classification results [20,30,39].

## 5. Conclusions

MRI-based radiomic features have the potential to serve as promising imaging biomarkers for discriminating between Warthin’s tumors and malignant tumors in the parotid gland. Nevertheless, it is still necessary to prove how radiomic features can consistently achieve high diagnostic performance, and if they can outperform alternative imaging or clinical models, ideally in larger, multicentric studies.

## Figures and Tables

**Table 1:** Distribution of parotid gland tumors.

Tumor Histology	Number (%)
Warthin’s tumors	66 (62.26)
Malignant parotid gland tumors	40 (37.74)
Squamous cell carcinomas	8 (7.55)
Metastatic tumors	8 (7.55)
Salivary duct carcinomas	6 (5.66)
Adenoid cystic carcinomas	6 (5.66)
Acinic cell carcinomas	4 (3.77)
Mucoepidermoid carcinomas	3 (2.83)
Basal cell carcinomas	2 (1.89)
Undifferentiated sarcomas	1 (0.94)
Histiocytic sarcomas	1 (0.94)
Carcinoma ex pleomorphic adenomas	1 (0.94)

**Table 2:** Radiomic features categories.

Images Type	Radiomic Feature Category	Number
Original	Shape	14
	First Order	18
	Second Order	
	GLCM (gray-level co-occurrence matrix)	24
	GLRLM (gray-level run-length matrix)	16
	GLSZM (gray-level size zone matrix)	16
	GLDM (gray-level dependence matrix)	14
	NGTDM (neighboring gray-tone difference matrix)	5
Filtered	Wavelet	744
	Laplacian of Gaussian	
	sigma 3.0 mm: 93	93
	sigma 5.0 mm: 93	93

**Table 3:** Non-redundant radiomic parameters able to discriminate between Warthin’s tumors and malignant tumors of the parotid gland.

MRI Sequence	Radiomic Feature Name	Radiomic Group	Associated Filter	*p*-Value *
T2-WI	JointEnergy	GLCM	LoG filter (3 mm)	<0.001
T2-WI	SmallAreaEmphasis	GLSZM	original	0.001
T2-WI	HighGrayLevelRunEmphasis	GLRLM	wavelet-LLH	0.013
T2-WI	JointAverage	GLCM	wavelet-LLL	0.002
T2-WI	GrayLevelNonUniformityNormalized	GLSZM	wavelet-HLH	0.022
T2-WI	Strength	NGTDM	LoG filter (5 mm)	0.012
T2-WI	Correlation	GLCM	wavelet-LHL	<0.001
T2-WI	GrayLevelVariance	GLSZM	LoG filter (3 mm)	0.015
T2-WI	SizeZoneNonUniformityNormalized	GLSZM	LoG filter (5 mm)	0.029
T2-WI	InterquartileRange	first order	wavelet-LLH	<0.001
T2-WI	Imc2	GLCM	LoG filter (3 mm)	0.019
T2-WI	Correlation	NGTDM	wavelet-LLH	0.016
T2-WI	RunEntropy	first order	wavelet-LLH	0.022
T2-WI	JointAverage	GLCM	original	0.005
T2-WI	Busyness	NGTDM	LoG filter (3 mm)	<0.001
ADC map	Contrast	GLCM	LoG filter (5 mm)	0.015
ADC map	RobustMeanAbsoluteDeviation	First order	LoG filter (3 mm)	0.029
ADC map	InverseVariance	GLCM	wavelet-LHH	0.011
ADC map	RootMeanSquared	first order	wavelet-HLL	<0.001
ADC map	SmallDependenceEmphasis	GLDM	LoG filter (5 mm)	0.038

T2-WI = T2-weighted image; ADC = apparent diffusion coefficient; GLCM = gray-level co-occurrence matrix; GLSZM = gray-level size zone matrix; GLRLM = gray-level run length matrix; NGTDM = neighboring gray-tone difference matrix; GLDM = gray-level dependence matrix; LoG = Laplacian of Gaussian. * *p*-value in the univariate analysis using the Mann–Whitney U test and the Benjamani–Hochberg correction for multiple tests.

**Table 4:** Radiomic parameters selection results following LASSO regression.

MRI Sequence	Radiomic Feature Name	Radiomic Group	Associated Filter	Coefficient
T2-WI	GrayLevelVariance	Texture GLSZM	LoG filter (3 mm)	−0.765
ADC map	RootMeanSquared	First order	Wavelet-HLL	0.24
T2-WI	HighGrayLevelRunEmphasis	Texture GLRLM	Wavelet-LLH	1.654
	Intercept			−0.895

T2-WI = T2-weighted image; ADC = Attenuated Diffusion Coefficient; glszm = gray-level size zone matrix; glrlm = gray-level run length matrix; LoG = Laplacian of Gaussian.

**Table 5:** Individual diagnostic performance of the final selected radiomic features.

Radiomic Feature	Cut-Off	Se (95% CI)	Sp (95% CI)	+LR (95% CI)	−LR (95% CI)	AUC (95% CI)	*p*
GrayLevelVariance	≤−0.488	67.74 (48.6–83.3)	81.25 (67.4–91.1)	3.61 (1.91–6.83)	0.40 (0.23–0.67)	0.767 (0.639–0.840	<0.001
RootMeanSquared	≤−0.142	77.42 (58.9–90.4)	64.58 (49.5–77.8)	2.19 (1.43–3.35)	0.35 (0.18–0.69)	0.738 (0.627–0.830)	<0.001
HighGrayLevelRunEmphasis	≥0.483	77.42 (58.9–90.4)	62.5 (47.5–76)	2.06 (1.37–3.12)	0.36 (0.18–0.72)	0.703 (0.590–0.801)	0.002

The 95% confidence interval (CI) values are shown in parentheses. Se = sensitivity; Sp = specificity; +LR = positive likelihood ratio; −LR = negative likelihood ratio; AUC = area under curve; *p* = statistical significance level.

**Table 6:** Diagnostic performance of the Radiomic Score in the training and testing datasets.

Radiomic Score	Cut-Off	Se (95% CI)	Sp (95% CI)	+LR (95% CI)	−LR (95% CI)	AUC (95% CI)	*p*
Training set	<−0.477	74.19 55.4–88.1	81.25 67.4–91.1	3.96 2.12–7.39	0.32 0.17–0.59	0.785 0.677–0.868	<0.001
Testing set	<−0.543	70 34.8–82.35	82.35 56.6–96.2	3.97 1.31–11.97	0.36 0.14–0.96	0.741 0.538–0.889	0.023

The 95% confidence interval (CI) values are shown in parentheses. Se = sensitivity; Sp = specificity; +LR = positive likelihood ratio; −LR = negative likelihood ratio; AUC = area under curve; *p* = statistical significance level.

## Data Availability

The data presented in this study are available in this article.

## Abbreviations

The following abbreviations are used in this manuscript:

PGT	parotid gland tumors
WT	Warthin’s tumor
MT	Malignant tumor
T2-WI	T2-weighted image
ADC	Apparent Diffusion Coefficient
AUC	Area Under the Curve

## References

[1] Stenner M., Klussmann J.P. (2009). Current update on established and novel biomarkers in salivary gland carcinoma pathology and the molecular pathways involved. Eur. Arch. Otorhinolaryngol..

[2] To V.S.H., Chan J.Y.W., Tsang R.K.Y., Wei W.I. (2012). Review of salivary gland neoplasms. ISRN Otolaryngol..

[3] El-Naggar A.K., Chan J.K.C., Grandis J.R., Takata T., Slootweg P.J. (2017). WHO Classification of Head and Neck Tumours.

[4] Bussu F., Parrilla C., Rizzo D., Almadori G., Paludetti G., Galli J. (2011). Clinical approach and treatment of benign and malignant parotid masses, personal experience. Acta Otorhinolaryngol. Ital..

[5] Tartaglione T., Botto A., Sciandra M., Gaudino S., Danieli L., Parrilla C., Paludetti G., Colosimo C. (2015). Differential diagnosis of parotid gland tumours: Which magnetic resonance findings should be taken in account?. Acta Otorhinolaryngol. Ital..

[6] Cracchiolo J.R., Shaha A.R. (2016). Parotidectomy for parotid cancer. Otolaryngol. Clin. N. Am..

[7] Zbären P., Triantafyllou A., Devaney K.O., Poorten V.V., Hellquist H., Rinaldo A., Ferlito A. (2018). Preoperative diagnostic of parotid gland neoplasms: Fine-needle aspiration cytology or core needle biopsy?. Eur. Arch. Otorhinolaryngol..

[8] Jering M., Mayer M., Thölken R., Schiele S., Maccagno A., Zenk J. (2022). Diagnostic Accuracy and Post-Procedural Complications Associated with Ultrasound-Guided Core Needle Biopsy in the Preoperative Evaluation of Parotid Tumors. Head Neck Pathol..

[9] Gökçe E. (2020). Multiparametric Magnetic Resonance Imaging for the Diagnosis and Differential Diagnosis of Parotid Gland Tumors. J. Magn. Reson. Imaging.

[10] Maraghelli D., Pietragalla M., Cordopatri C., Nardi C., Peired A.J., Maggiore G., Colagrande S. (2021). Magnetic resonance imaging of salivary gland tumours: Key findings for imaging characterisation. Eur. J. Radiol..

[11] Pietragalla M., Nardi C., Bonasera L., Mungai F., Taverna C., Novelli L., De Renzis A.G.D., Calistri L., Tomei M., Occhipinti M. (2020). The role of diffusion-weighted and dynamic contrast enhancement perfusion-weighted imaging in the evaluation of salivary glands neoplasms. Radiol. Med..

[12] Coudert H., Mirafzal S., Dissard A., Boyer L., Montoriol P.F. (2021). Multiparametric magnetic resonance imaging of parotid tumors: A systematic review. Diagn. Interv. Imaging.

[13] Ikeda M., Motoori K., Hanazawa T., Nagai Y., Yamamoto S., Ueda T., Funatsu H., Ito H. (2004). Warthin tumor of the parotid gland: Diagnostic value of MR imaging with histopathologic correlation. Am. J. Neuroradiol..

[14] Nguyen V.X., Nguyen B.D., Ram P.C. (2012). Bilateral and multifocal Warthin's tumors of parotid glands: PET/CT imaging. Clin. Nucl. Med..

[15] Gillies R.J., Kinahan P.E., Hricak H. (2016). Radiomics: Images are more than pictures, they are data. Radiology.

[16] Aringhieri G., Fanni S.C., Febi M., Colligiani L., Cioni D., Neri E. (2022). The Role of Radiomics in Salivary Gland Imaging: A Systematic Review and Radiomics Quality Assessment. Diagnostics.

[17] Zhang R., Ai Q.Y.H., Wong L.M., Green C., Qamar S., So T.Y., Vlantis A.C., King A.D. (2022). Radiomics for Discriminating Benign and Malignant Salivary Gland Tumors; Which Radiomic Feature Categories and MRI Sequences Should Be Used?. Cancers.

[18] Hu Z., Guo J., Feng J., Huang Y., Xu H., Zhou Q. (2023). Value of T2-weighted-based radiomics model in distinguishing Warthin tumor from pleomorphic adenoma of the parotid. Eur. Radiol..

[19] Gabelloni M., Faggioni L., Attanasio S., Vani V., Goddi A., Colantonio S., Germanese D., Caudai C., Bruschini L., Scarano M. (2020). Can Magnetic Resonance Radiomics Analysis Discriminate Parotid Gland Tumors? A Pilot Study. Diagnostics.

[20] Wen B., Zhang Z., Zhu J., Liu L., Li Y., Huang H., Zhang Y., Cheng J. (2022). Apparent Diffusion Coefficient Map-Based Radiomics Features for Differential Diagnosis of Pleomorphic Adenomas and Warthin Tumors from Malignant Tumors. Front. Oncol..

[21] Kocak B., Baessler B., Bakas S., Cuocolo R., Fedorov A., Maier-Hein L., Mercaldo N., Müller H., Orlhac F., Pinto Dos Santos D. (2023). CheckList for EvaluAtion of Radiomics research (CLEAR): A step-by-step reporting guideline for authors and reviewers endorsed by ESR and EuSoMII. Insights Imaging.

[22] Shur J.D., Doran S.J., Kumar S., Ap Dafydd D., Downey K., O’Connor J.P.B., Papanikolaou N., Messiou C., Koh D.M., Orton M.R. (2021). Radiomics in Oncology: A Practical Guide. Radiographics.

[23] Zhang Z., Song C., Zhang Y., Wen B., Zhu J., Cheng J. (2019). Apparent diffusion coefficient (ADC) histogram analysis: Differentiation of benign from malignant parotid gland tumors using readout-segmented diffusion-weighted imaging. Dentomaxillofacial Radiol..

[24] Wada T., Yokota H., Horikoshi T., Starkey J., Hattori S., Hashiba J., Uno T. (2020). Diagnostic performance and inter-operator variability of apparent diffusion coefficient analysis for differentiating pleomorphic adenoma and carcinoma ex pleomorphic adenoma: Comparing one-point measurement and whole-tumor measurement including radiomics approach. Jpn. J. Radiol..

[25] Tustison N.J., Avants B.B., Cook P.A., Zheng Y., Egan A., Yushkevich P.A., Gee J.C. (2010). N4ITK: Improved N3 bias correction. IEEE Trans. Med. Imaging.

[26] Collewet G., Strzelecki M., Mariette F. (2004). Influence of MRI acquisition protocols and image intensity normalization methods on texture classification. Magn. Reson. Imaging.

[27] Zwanenburg A., Vallières M., Abdalah M.A., Aerts H.J.W.L., Andrearczyk V., Apte A., Ashrafinia S., Bakas S., Beukinga R.J., Boellaard R. (2020). The Image Biomarker Standardization Initiative: Standardized Quantitative Radiomics for High-Throughput Image-based Phenotyping. Radiology.

[28] Depeursinge A., Foncubierta-Rodriguez A., Van De Ville D., Müller H. (2014). Three-dimensional solid texture analysis in biomedical imaging: Review and opportunities. Med. Image Anal..

[29] Ganeshan B., Miles K.A. (2013). Quantifying tumour heterogeneity with CT. Cancer Imaging.

[30] Zheng Y.M., Li J., Liu S., Cui J.F., Zhan J.F., Pang J., Zhou R.Z., Li X.L., Dong C. (2021). MRI-Based radiomics nomogram for differentiation of benign and malignant lesions of the parotid gland. Eur. Radiol..

[31] He Z., Mao Y., Lu S., Tan L., Xiao J., Tan P., Zhang H., Li G., Yan H., Tan J. (2022). Machine learning-based radiomics for histological classification of parotid tumors using morphological MRI: A comparative study. Eur. Radiol..

[32] Ren J., Yuan Y., Shi Y., Tao X. (2019). Tumor heterogeneity in oral and oropharyngeal squamous cell carcinoma assessed by texture analysis of CT and conventional MRI: A potential marker of overall survival. Acta Radiol..

[33] Shimamoto H., Tsujimoto T., Kakimoto N., Majima M., Iwamoto Y., Senda Y., Murakami S. (2018). Effectiveness of the periodically rotated overlapping parallel lines with enhanced reconstruction (PROPELLER) technique for reducing motion artifacts caused by mandibular movements on fat-suppressed T2-weighted magnetic resonance (MR) images. Magn. Reson. Imaging.

[34] Fruehwald-Pallamar J., Czerny C., Holzer-Fruehwald L., Nemec S.F., Mueller-Mang C., Weber M., Mayerhoefer M.E. (2013). Texture-based and diffusion-weighted discrimination of parotid gland lesions on MR images at 3.0 Tesla. NMR Biomed..

[35] Sarioglu O., Sarioglu F.C., Akdogan A.I., Kucuk U., Arslan I.B., Cukurova I., Pekcevik Y. (2020). MRI-based texture analysis to differentiate the most common parotid tumours. Clin. Radiol..

[36] Lobo R., Hawk J., Srinivasan A. (2018). A Review of Salivary Gland Malignancies: Common Histologic Types, Anatomic Considerations,and Imaging Strategies. Neuroimaging Clin..

[37] Cattell R., Chen S., Huang C. (2019). Robustness of radiomic features in magnetic resonance imaging: Review and a phantom study. Vis. Comput. Ind. Biomed. Art.

[38] van Timmeren J.E., Cester D., Tanadini-Lang S., Alkadhi H., Baessler B. (2020). Radiomics in medical imaging-“how-to” guide and critical reflection. Insights Imaging.

[39] Muntean D.D., Dudea S.M., Băciuț M., Dinu C., Stoia S., Solomon C., Csaba C., Rusu G.M., Lenghel L.M. (2023). The Role of an MRI-Based Radiomic Signature in Predicting Malignancy of Parotid Gland Tumors. Cancers.

